# An optimised protocol for isolation of RNA from small sections of laser-capture microdissected FFPE tissue amenable for next-generation sequencing

**DOI:** 10.1186/s12867-017-0099-7

**Published:** 2017-08-23

**Authors:** Parisa Amini, Julia Ettlin, Lennart Opitz, Elena Clementi, Alexandra Malbon, Enni Markkanen

**Affiliations:** 1 Institute of Veterinary Pharmacology and Toxicology, Vetsuisse Faculty, University of Zürich, Winterthurerstr. 260, 8057 Zurich, Switzerland; 2 Functional Genomics Center Zurich, University of Zürich/ETH Zürich, Winterthurerstr. 190, 8057 Zurich, Switzerland; 3 Institute of Veterinary Pathology, Vetsuisse Faculty, University of Zürich, Winterthurerstr. 268, 8057 Zurich, Switzerland

**Keywords:** FFPE, RNA isolation, RNAsequencing, LCM, RT-qPCR, Dog, Cancer, Mammary carcinoma, Mammary tumour

## Abstract

**Background:**

Formalin-fixed paraffin embedded (FFPE) tissue constitutes a vast treasury of samples for biomedical research. Thus far however, extraction of RNA from FFPE tissue has proved challenging due to chemical RNA–protein crosslinking and RNA fragmentation, both of which heavily impact on RNA quantity and quality for downstream analysis. With very small sample sizes, e.g. when performing Laser-capture microdissection (LCM) to isolate specific subpopulations of cells, recovery of sufficient RNA for analysis with reverse-transcription quantitative PCR (RT-qPCR) or next-generation sequencing (NGS) becomes very cumbersome and difficult.

**Methods:**

We excised matched cancer-associated stroma (CAS) and normal stroma from clinical specimen of FFPE canine mammary tumours using LCM, and compared the commonly used protease-based RNA isolation procedure with an adapted novel technique that additionally incorporates a focused ultrasonication step.

**Results:**

We successfully adapted a protocol that uses focused ultrasonication to isolate RNA from small amounts of deparaffinised, stained, clinical LCM samples. Using this approach, we found that total RNA yields could be increased by 8- to 12-fold compared to a commonly used protease-based extraction technique. Surprisingly, RNA extracted using this new approach was qualitatively at least equal if not superior compared to the old approach, as Cq values in RT-qPCR were on average 2.3-fold lower using the new method. Finally, we demonstrate that RNA extracted using the new method performs comparably in NGS as well.

**Conclusions:**

We present a successful isolation protocol for extraction of RNA from difficult and limiting FFPE tissue samples that enables successful analysis of small sections of clinically relevant specimen. The possibility to study gene expression signatures in specific small sections of archival FFPE tissue, which often entail large amounts of highly relevant clinical follow-up data, unlocks a new dimension of hitherto difficult-to-analyse samples which now become amenable for investigation.

**Electronic supplementary material:**

The online version of this article (doi:10.1186/s12867-017-0099-7) contains supplementary material, which is available to authorized users.

## Introduction

Formalin-fixed paraffin-embedded (FFPE) tissue samples constitute a vast and valuable resource of patient material that can potentially be used for biomedical research. In most cases such specimen also entail large amounts of clinically relevant data, such as clinical history, further laboratory findings, follow-up data, and much more. To date however, the extraction of macromolecules in general, and RNA in particular, from FFPE tissues has proved challenging due to chemical crosslinking of RNA with proteins and RNA fragmentation, both of which heavily decrease RNA quantity that can be extracted from such tissues, and also severely impact on RNA quality for downstream analysis (e.g. [1–4]).

Laser-capture microdissection (LCM) is a technique that allows the specific isolation of defined areas, such as particular subpopulations of cells, from within a tissue section by direct microscopic visualization [5]. This approach enables researchers to precisely dissect areas of interest and thus address the role(s) of specific subsets of cells within samples of a given pathology directly derived from patients. Such an approach is particularly valuable and important as the focus of research is switching towards deciphering the cellular and molecular interactions of different contributing cell types that underlie pathologies. For instance, as data from the tumour research field convincingly demonstrates, cancerous lesions are not only made up of neoplastic tumour cells, but can be rather thought of as an ‘organism’ in which vast contributions to support survival and proliferation derive from the surrounding microenvironment that consists of stromal cells, immune cells, tumour vasculature, extracellular matrix and other components [6, 7]. The problem that arises when addressing small subsections of tissue sections that are excised by LCM is often one of low abundance—the sections that can be isolated are more often than not very small, thus also yielding very low amounts of material to study. If, in addition to this complication, the isolated material has a rather low cellularity, such as stroma that consists in large parts of extracellular matrix, isolation of sufficient RNA can become a really taunting challenge.

Due to all these facts, to date most LCM analyses geared towards RNA expression analysis have used fresh-frozen rather than FFPE tissue sections [1]. However, the use of fresh-frozen tissues necessitates a high grade of coordination between surgical tissue resection and the analysis pipeline, which often proves difficult, and importantly also precludes the analysis of any archival samples that might be available. Furthermore, tissue morphology of FFPE tissue is vastly superior to fresh-frozen tissue, and staining procedures for specific subpopulations of cells are often more optimal for FFPE tissue. Therefore, a method that would allow better use of FFPE tissue for RNA expression analysis has the potential to benefit a vast array of research projects that are analysing patient-derived specimen.

We have recently established a procedure to extract cancer-associated stroma (CAS) from archival canine FFPE breast cancer tissue by LCM using reverse-transcription quantitative PCR (RT-qPCR) [8]. During the establishment of this procedure, a major problem we encountered was the low overall yield of RNA that could be extracted from the tiny amounts of tissue that were isolated, which rendered the tissue sampling and analysis slow and cumbersome, if not almost impossible. To be able to tap the valuable reserve of FFPE tissue specimen for analysis by LCM efficiently, we felt that a better protocol for RNA isolation could help improving the yields of RNA that is isolated from these tissues. To this end, we set out to improve the extraction of RNA from these very small and challenging deparaffinised and stained FFPE samples with the ultimate aim of performing next-generation sequencing (NGS) RNA analysis.

## Materials and methods

### Case selection and tissue processing for LCM

Thirteen dog mammary carcinoma samples were obtained from the Institute of Veterinary Pathology of the Vetsuisse Faculty Zürich (Table 1). All samples were formalin-fixed, paraffin-embedded tissue samples either from the Small Animal Hospital of Zurich or external cases sent in by veterinarians practising in Switzerland. Details regarding selection criteria are described in [8]. Paraffin blocks were routinely kept at room temperature. For LCM, tissue sections were cut at 10 µm. DEPC treated water was used for the microtome HM 360 (ThermoFisher Scientific), the blade was cleaned with RNase away™ (Ambion). The tissue was mounted on PEN Membrane Glass Slides (Applied Biosystems™) and mounted tissue sections were left to dry overnight at room temperature (http://support.moleculardevices.com). To visualise the areas of interest, tissue sections were stained with Cresyl Fast Violet according to [5] with slight modifications (Table 2). To allow for proper excision performance, slides were completely air dried before microdissection. For every tissue sample that underwent LCM, a second tissue slide was stained with conventional Hematoxylin–Eosin staining to allow for validation of tissue morphology in case of uncertainty using the Cresy violet stain.

**Table 1:** Overview of cases included in this study

Case #	Gender	Breed	Age (years)	Subtype of simple carcinoma
1	f	Basset	12	Tubular
2	f	Vizsla	10	Cystic-papillary
3	f	Samoyed	5	Tubulo-papillary
4	f	Maltese	14	Tubular
5	f	Tibetan terrier	12	Tubular
6	f/n	West highland white terrier	12	Tubular-solid
7	f	Havanese	13	Tubular
8	f	Chihuahua	8	Tubulo-papillary
9	f/n	Bracke	9	Cribriform
10	f/n	N.d.	13	Tubular
11	f/n	Appenzell mountain dog	6	Tubular
12	f	Boxer	9	Tubulo-papillary
13	f	N.d.	4	Cystic-papillary

Clinical data from dogs with simple mammary carcinoma; Case# case number as referred to within this study, f/n female, neutered, n.d not disclosed. Age age at excision of tumour

**Table 2:** Protocol for Cresyl violet staining of FFPE tissue sections

Cresyl violet staining procedure for FFPE tissue sections	
100% Xylene, bath 1	5 min
100% Xylene, bath 2	5 min
100% Ethanol	30 s
95% Ethanol	30 s
70% Ethanol	30 s
dH_2_O	10 s
Cresyl violet (75% Ethanol with DEPC treated dH_2_O, pH 8.0)	15 s
dH_2_O	10 s
70% Ethanol	10 s
95% Ethanol, bath 1	10 s
95% Ethanol, bath 2	10 s
100% Ethanol, bath 1	30–60 s
100% Ethanol, bath 2	30–60 s

### Laser-capture microdissection (LCM)

**Fig. 1:**
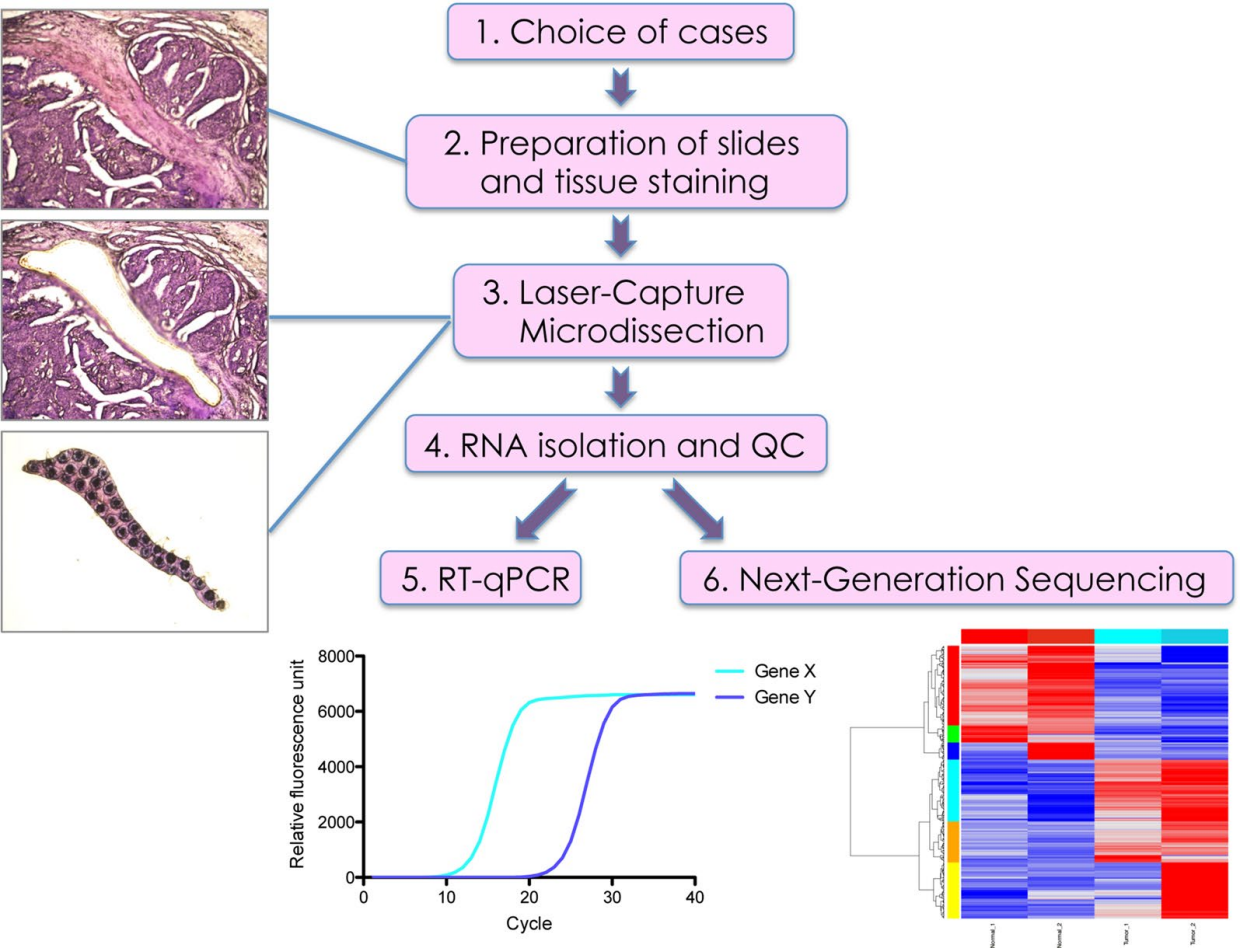
Workflow for the isolation and analysis of RNA from normal and cancer-associated stroma from FFPE tissue by Laser-capture microdissection. (*1*) Canine simple mammary carcinoma cases to be analysed are chosen from the archives. (*2*) Sectioning and mounting of FFPE sections is followed by tissue deparaffinisation and staining to visualise structures under the microscope (*top panel on the left*). (*3*) Laser-capture microdissection allows excision of areas of interest, and visual validation of the excised area (*middle panel on the left*) as well as the excised tissue piece (*bottom panel on the left*). The black spots that appear on the excised tissue piece are a result of the thermoplastic film that is welded to the tissue, and not tissue damage. (*4*) RNA is isolated from the excised tissue of interest, and quality control is performed to measure RNA quality and quantity. (*5*) RT-qPCR or (*6*) NGS can be used to analyse the extracted RNA

Before microdissection, identification of tumour stroma in samples was performed by a pathologist (Alexandra Malbon AM). Criteria for stroma: fibroblastic cells, endothelial cells and pericytes of small vessels, only single inflammatory cells to avoid areas with heavy inflammation, no adipocytes. For Microdissection the ArcturusXT™ Laser Capture Microdissection System (Thermo Scientific) and Arcturus^®^ CapSure^®^ Macro LCM Caps (Life Technologies) were used. Highly enriched populations of normal or tumour-associated stroma from the specimen were identified and isolated according to the manufacturer’s protocol. Normal stroma samples were isolated from the same slides, from regions specified by a pathologist (AM) that presented no obvious alterations or at least 2 mm away from the tumour [9]. Isolation of cells of interest was verified by microscopic examination of the LCM cap as well as the excised region after microdissection (Fig. 1). After excision, the caps were put on 0.5 ml microcentrifuge tubes (Eppendorf^®^ Safe-Lock Tubes) and placed on ice until proceeding with mRNA isolation. 1–2 caps were used per sample.

### **‘**Old’ (protease-based) isolation protocol for RNA from FFPE tissue sections

Extraction of mRNA was performed immediately after microdissection using the Recover All™ Total Nucleic Acid Isolation Kit for FFPE (Ambion™) according to the manufacturer’s protocol with the following small adjustments. As long exposure to xylene has been shown to be detrimental to mRNA integrity [10], and deparaffinisation by xylene had already been performed to stain the sections, the first deparaffinisation step using xylene and 100% ethanol was skipped and the excised tissue was directly immersed into a 0.5 ml microcentrifuge tube containing 100 µl Digestion Buffer and 4 µl Protease. To get the tissue into the solution a sterile blade and forceps were used to peel off the thermoplastic film from the cap containing the captured cells. The heating time and temperature in step C-2a was adjusted to 3 h at 50 °C followed by 20 min at 70 °C, according to manufactures protocol “Optimized Extraction and Quantification of RNA from FFPE Samples for Gene Expression Analyses” (https://tools.thermofisher.com). To elute the RNA from the column, RNase-free water was used to avoid the effects of elution buffer on downstream applications. The eluate was aliquoted before analysis and stored at −80 °C. RNA abundance and quality was analysed using the 4200 or 2200 Tape Station Software using the High Sensitivity RNA ScreenTape kit (Agilent Technologies), according to the manufacturer’s protocol.

### **‘**New’ (sonication-based) isolation protocol for RNA from FFPE tissue sections

Extraction of RNA was performed immediately after microdissection using the Covaris^®^ truXTRAC FFPE RNA kit according to the manufacturer’s protocol with following adjustments. A sterile blade was used to peel off the thermoplastic film from the LCM cap and transfer the tissue isolated by LCM into glass vials for sonication. Sonication of samples was performed with the E220 focused ultrasonicator (Covaris^®^). After reverse crosslinking at 80 °C, the soluble fraction was transferred into clean eppendorf tubes and DNAse treated without prior centrifugation, as due to the absence of paraffin, the samples did not contain any solids that could be precipitated. RNA was eluted from the spin columns using 30 µl of elution buffer prewarmed to 70 °C to increase RNA yield. A second elution into fresh collection tubes was performed using 20–30 µl prewarmed elution buffer using the identical elution protocol. The eluate was aliquoted before analysis and stored at −80 °C. RNA abundance and quality was analysed using the 4200 or 2200 Tape Station Software using the High Sensitivity RNA ScreenTape kit (Agilent Technologies), according to the manufacturer’s protocol.

### cDNA Synthesis, preamplification and quantitative real-time PCR

Reverse transcription was performed using the iScript™ cDNA Synthesis Kit (BioRad) according to the manufacturer’s protocol, using a maximum of 10 µl of RNA per reaction. Due to the limiting concentration extractions using the old method, RNA inputs per reaction ranged between 0.5 and 2.3 ng of total RNA per sample. This kit allows generation of cDNA with combination of oligo(dT) and random hexamer primers using low RNA inputs and is optimized for fragments below 1 kb of length. To increase the number of RT-qPCR analyses that could be performed, cDNA was preamplified using the TaqMan^®^ PreAmp Master Mix (2×) (Applied Biosystems™). This step was necessary due to the very low concentrations of RNA extracted using the old protocol, but can be omitted using the new protocol that yields much higher RNA amounts and concentration. For comparability reasons it was performed identically in parallel for all samples. The preamplification was performed according to the manufacturer’s protocol using 14 PCR cycles. RT-qPCR was performed using KAPA PROBE FAST qPCR Kit Master Mix (2×) Universal reagents (Kapa Biosystems), with 2.5 µl cDNA per reaction, in a total volume of 10 µl. RT-qPCR were run in duplicates on the CFX384 Touch™ Real-Time PCR detection system (BioRad). Details regarding primers can be found in [8], and other details regarding the setup of the RT-qPCR can be found in [11].

### Next-generation sequencing

RNA library preparation and depletion of ribosomal RNA was performed using the SMARTer Stranded Total RNA-seq Kit—Pico Input Mammalian from Clontech/Takara Bio USA according to the manufacturer’s protocol with 2 ng input RNA for the ‘old’ extraction protocol, and 10 ng input RNA for the ‘new’ extraction protocol. Single-read sequencing (125 bp) was run on the Illumina HiSeq 2500 using the HiSeq SBS Kit v4 and HiSeq cluster kits v4 according to standard protocols used at the Functional Genomics Centre Zurich (FGCZ). Resulting NGS reads were quality-checked with FastQC (http://www.bioinformatics.babraham.ac.uk/projects/fastqc). Reads were trimmed with Trimmomatic [12] (v0.33, 4 bases hard-trimming from the start, and adapter trimming at the end). We aligned the trimmed reads to the reference genome and transcriptome (FASTA and GTF files, respectively, Ensembl, release88, CanFam3.1) with STAR [13] version 2.5.1b. Gene expression was quantified using the R/Bioconductor package Rsubread (version 1.24.1) [14]. To detect differentially expressed genes, we applied the count based negative binomial model implemented in the R/Bioconductor package edgeR (R version: 3.3.2, edgeR version: 3.16.5) [15], in which the normalization factor was calculated by trimmed mean of M values (TMM) method [16]. The sequence data of this study have been deposited in the European Nucleotide Archive with the primary accession code PRJEB20761.

### Graphical display of results and statistical analysis

For all statistical analysis and graphical display the program GraphPad Prism (http://www.graphpad.com) was used.

## Results

### Workflow for the isolation of RNA from normal and cancer-associated stroma from FFPE tissue

To specifically isolate normal stroma and CAS from within a section of FFPE tissue, we had previously established a workflow for case selection, tissue preparation and staining, LCM, and isolation of RNA, which can be followed by RNA analysis by either RT-qPCR or NGS (Fig. 1) and [8]. We used the ArcturusXT™ Laser Capture Microdissection System (Thermo Scientific) to isolate matched normal and CAS from 13 clinical cases of canine simple mammary carcinoma (Table 1). Of note, to minimise differences in tissue quality and processing, normal stroma and CAS were both isolated from the identical tissue section. This allows optimal comparability of the two correlates. The areas to be isolated were defined by a board-certified veterinary pathologist (AM), and microscopic validation of tissue before and after excision, as well as the excised portion, ensured selective isolation of the tissue of interest (Fig. 1). RNA isolation, performed as specified later, was followed by control of RNA quality and quantity as described in “Materials and methods” section.

### Optimization of the RNA isolation procedure

**Fig. 2:**
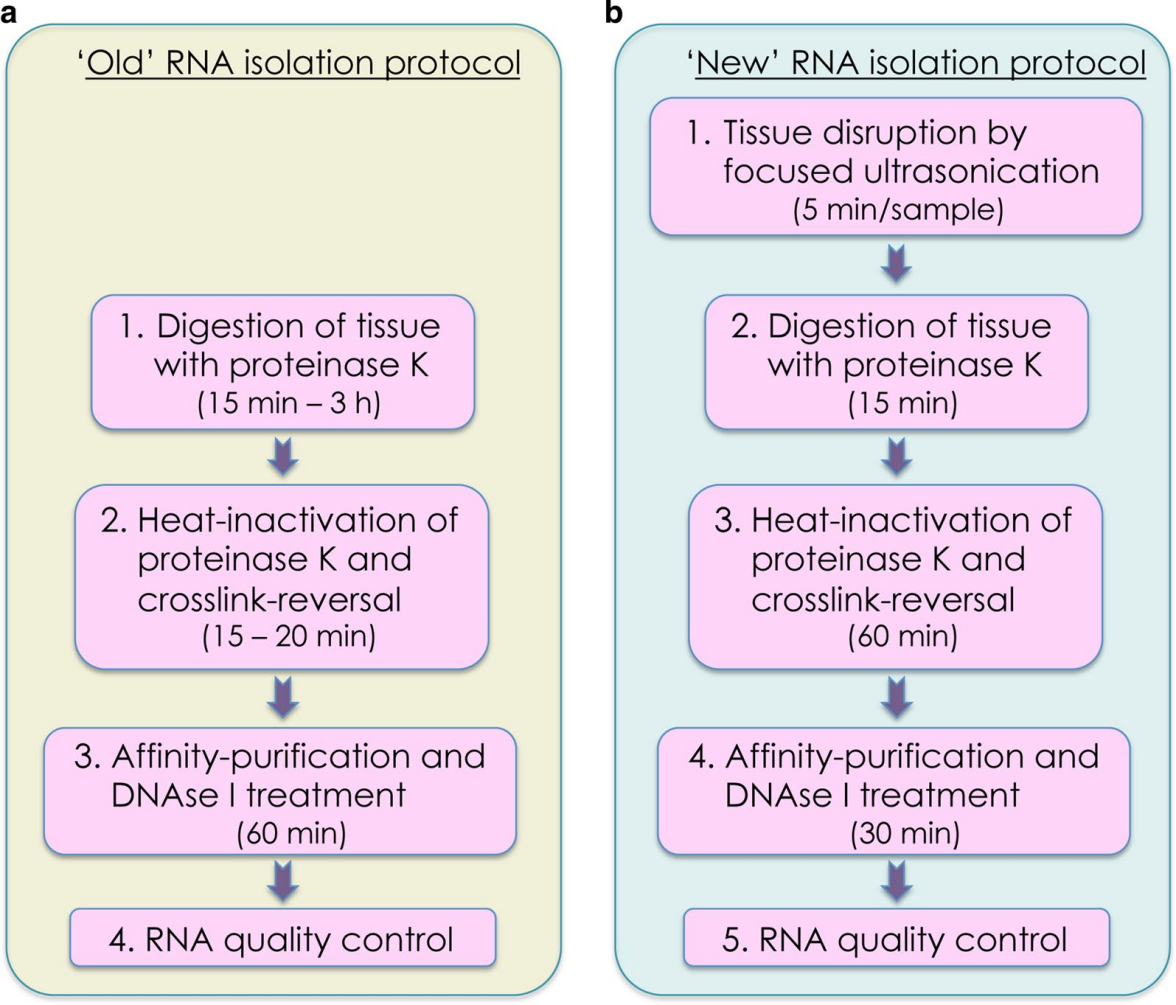
Comparison of workflow of the ‘old’(classical) and ‘new’ protocol for RNA isolation from deparaffinised, stained and laser-capture microdissected FFPE tissue. **a** Workflow of the ‘old’ RNA isolation protocol, starting with proteinase K digestion of the sample. **b** Workflow of the ‘new’ isolation protocol, starting with a focused ultrasonication step to disrupt the tissue prior to proteinase K digestion. *Values in brackets* indicate the approximate timing for each step

Most classically available protocols and kits for extraction of RNA from FFPE tissue depend entirely on a Proteinase K (or another equivalent protease) digestion step to lyse the tissue and liberate RNA (Fig. 2a), usually following deparaffinisation of the sample (e.g. [17–21]). Proteinase K digests proteins, which leads to unravelling of the tissue, and also removes at least a part of the proteins from protein–RNA crosslinks that are the result of formalin fixation [22]. This digestion step is usually followed by a heating step to inactivate the enzyme and further promote the reversal of protein-RNA crosslinks. The resulting mixture of macromolecules is then treated with DNAse I to remove unwanted DNA, and cleaned up using an affinity purification step, often consisting of a spin-column, or similar.

**Fig. 3:**
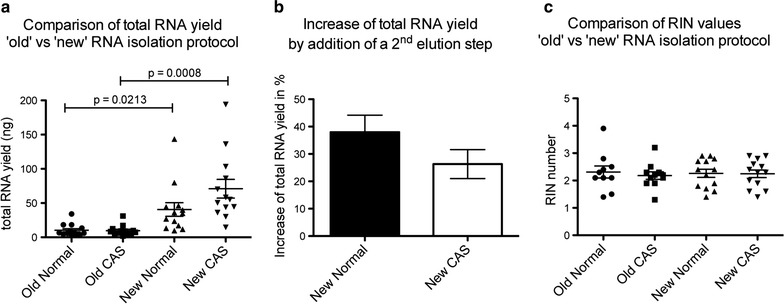
Direct comparison of RNA extracted from LCM samples from deparaffinised, stained FFPE tissue with the old vs the new isolation protocol. **a** Scatter plot with mean ± SEM of total RNA yield from the 13 clinical cases analysed obtained using the old vs the new isolation protocol. Old normal shows data obtained from using the old protocol on LCM samples of normal stroma, while old CAS shows data for the old protocol on CAS. New normal and new CAS are the equivalent for the new extraction procedure, respectively. p-values were calculated using the Student’s t test. **b** Bar graph displaying mean ± SEM of the increase of total RNA yield that could be achieved using a 2nd elution step with the new isolation protocol for normal and CAS samples, respectively, compared to a single elution step only. n = 8 for both types of tissue, respectively. **c** Scatter plot with mean ± SEM of the RIN values obtained from all 13 clinical cases isolated using the old or the new isolation procedure, respectively. RIN values could not be calculated for 3 samples of old normal and 2 samples of old CAS, and are thus omitted from this graph

In our experiments, the major bottleneck of RNA extraction using the classical approach seemed to derive from the inefficiency of Proteinase K to completely digest the very fibrous tissue (CAS and normal stroma) that had been isolated by LCM. Despite trying marked increases in digestion time (as suggested by several available protocols, e.g. [17, 20]), we could still observe clearly macroscopically visual pieces of tissue that would not dissolve, and most likely still contained substantial amounts of RNA that remained inaccessible to our extraction efforts, contributing to low total RNA yields (Fig. 3a; Additional file 1: Table S1 and [8]).

To overcome this problem, we decided to test a novel approach for isolation of RNA from FFPE tissue, which relies on tissue disruption using focused ultrasonication prior to the proteinase K treatment step (Fig. 2b). The main problem was that this approach has been developed for use with paraffinised tissue sections exclusively, and was not supposed to work with deparaffinised and stained tissue. For paraffinised tissue, the ultrasonication step leads to an emulsification of the paraffin with the lysis solution, which helps removing paraffin from the sample, improves tissue rehydration, and enhances dissociation of biomolecules for improved isolation of RNA [23, 24]. However, deparaffinisation is a prerequisite for tissue staining in order to properly visualise tissue for LCM. Nevertheless, we reasoned that tissue disruption using focused sonication should still be efficient even in the absence of paraffin, and adapted the workflow of the kit slightly to suit our samples (for details see "Materials and methods"). Apart from the first tissue disruption step, this novel RNA isolation protocol is very comparable to the other available kits, also in terms of hands-on time (compare Fig. 2a, b).

### Direct comparison of the old versus new FFPE RNA isolation protocol

To analyse the performance of the ‘new’ RNA isolation protocol, we isolated comparable amounts of normal stroma and CAS by LCM from the identical FFPE tissue blocks that had been used for our previous study, in which RNA had been isolated by the conventional ‘old’ method ([8] and Table 2). In order to keep the conditions as comparable as possible, we kept slide handling identical, and we sought to isolate similar surface areas visually and by using the same number of LCM caps (1–2 caps per sample) while also maintaining a similar timing for the entire process.

Comparing the results from both extraction methods, we observed a vast increase in RNA yield that could be isolated from the LCM specimen using the new isolation method (Fig. 3a; Additional file 1: Table S1). On average, the yield of RNA from normal stroma increased by fourfold from 10.18 ng (range 2.9–34 ng, Std. deviation 8.8 ng) using the old protocol to 40.42 ng (range 9.7–143.6 ng, Std. deviation 36.4 ng) using the new protocol, while the RNA yield from CAS increased by 7.4-fold from 9.57 ng (range 1.7–31 ng, Std. deviation 7.6 ng) with the old protocol to 70.95 ng (range 14.6–194 ng, Std. deviation 49.1 ng) using the new protocol. These numbers are however still underestimating the total increase of RNA yield that can be achieved using the new method, as we discovered in the course of the experiments that the addition of a second elution step yielded an additional 32.2% of RNA on average using the new protocol (38.0% (range 15.8–63.3%, Std. deviation 17.6%) more RNA from the normal stroma, and 26.3% (range 10.2–53.5%, Std. deviation 14.9%) more RNA from CAS) (Fig. 3b). In the four specimen included in this study where a 2nd elution step was performed, the average yield increased by 8.2-fold in the normal stroma (from 8.0 ng (range 3.3–18.2 ng, Std. deviation 6.9 ng) with the old protocol to 65.4 ng (range 17–143.6 ng, Std. deviation 59.4 ng) using the new protocol), and by 12.8-fold in the tumour stroma (from 8.2 ng (range 3.1–12.4 ng, Std. deviation 3.9 ng) with the old protocol to 104.6 ng (range 43.9–194 ng, Std. deviation 73.7 ng) using the new protocol). RNA integrity (RIN) values did not differ significantly between the extraction procedures (Fig. 3c; Additional file 1: Table S1).

### Comparison of the performance of the isolated RNA using RT-qPCR

To compare the performance of RNA extracted using the two methods by RT-qPCR, we randomly picked several of our samples, reverse transcribed identical amounts of RNA extracted using both methods into cDNA in parallel, and analysed expression of the two housekeeping genes GAPDH and B2M by RT-qPCR. RNA isolated using the new method performed in general better yielding lower mean Cq values in RT-qPCR than RNA deriving from the old isolation procedure (Table 3). On average, RNA from the new isolation procedure yielded lower mean Cq values by 2.06 cycles for GAPDH primers, and by 2.59 cycles for B2M primers compared to the mean Cq values using RNA from the old isolation protocol.

**Table 3:** Comparison of Cq values obtained using RNA isolated with the old vs. the new protocol

Primer	Sample	Mean Cq old	Mean Cq new	Δ Mean Cq new – mean Cq old
GAPDH	#2 Normal	26.99	26.56	−0.43
	#2 Tumour	24.89	24.61	−0.29
	#3 Tumour	22.24	17.75	−4.49
	#4 Tumour	22.99	19.23	−3.75
	#5 Normal	26.74	24.40	−2.34
	#6 Tumour	20.97	20.98	0.00
	#7 Tumour	23.57	19.79	−3.78
	#8 Normal	23.72	23.28	−0.44
	#9 Normal	27.36	27.77	0.41
	#9 Tumour	24.82	19.33	−5.49
	#10 Normal	27.29	26.89	−0.40
	#10 Tumour	N.d.	25.68	n.a.
	Mean ΔCq New−Cq Old for GAPDH			−2.06
B2M	#2 Normal	25.83	24.62	−1.21
	#2 Tumour	21.88	21.27	−0.61
	#3 Tumour	22.76	17.48	−5.28
	#4 Tumour	22.70	18.83	−3.87
	#5 Normal	24.33	21.38	−2.95
	#6 Tumour	21.32	21.07	−0.25
	#7 Tumour	23.89	18.87	−5.02
	#8 Normal	24.13	24.46	0.33
	#9 Normal	27.63	26.14	−1.49
	#9 Tumour	25.94	21.36	−4.58
	#10 Normal	28.98	26.73	−2.25
	#10 Tumour	27.92	24.16	−3.76
	Mean ΔCq New−ΔCq Old for B2M			−2.59

Identical amounts of RNA were reverse transcribed and analysed by RT-qPCR using GAPDH or B2 M primers. First column: primers that were used for the analysis. Second column indicates the case and type of tissue from which the RNA that was analysed derived. “Mean Cq Old” denotes the mean Cq values obtained with RNA isolated using the old extraction protocol. “Mean Cq New” shows the Cq values obtained with RNA isolated using the new extraction protocol. “Δ Mean Cq New-mean Cq old” shows the difference in Cq values between the new extraction protocol and the old extraction protocol; a negative value here indicates better performance (lower Cq values) of the new extraction protocol. “Mean Δ Cq New-Cq old values” for GAPDH and B2 M list the mean difference of the Cq values between new and old extraction across all samples. N.d not detectable, n.a not applicable.

### Comparison of the performance of the isolated RNA using Next-Generation RNA Sequencing

**Fig. 4:**
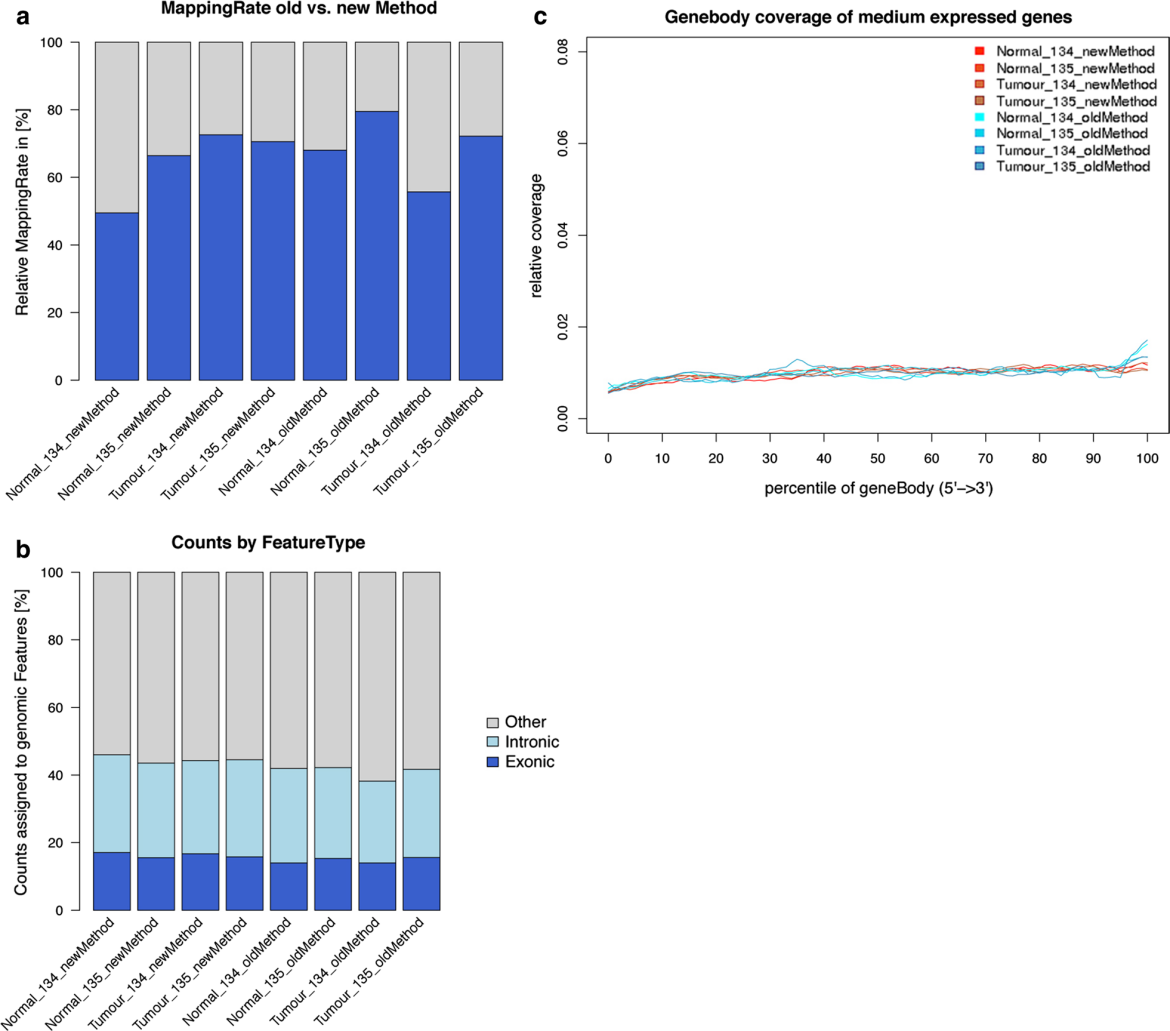
Comparison of Next-Generation Sequencing results obtained with RNA isolated with the old and new method, respectively. **a** Barplot illustrating the relative amount of mapped (*blue*) vs. unmapped (*grey*) reads using the new protocol (*left*) or the old protocol (*right*). **b** Barplot showing fractions of mapped reads specific for exonic (*blue*), intronic (*light blue*) and other genomic (*grey*) regions. **c** Linegraph illustrating the calculated read-coverage over the genebody for expressed genes of medium length (400–1000 nt). Results for samples extracted by the new protocol are highlighted in *red* colors

To compare the performance of the extracted RNA from both protocols using RNAseq, we checked the overall mapping rate for all generated reads. We observed that, regardless of the isolation protocol used, 49.45–79.51% of the reads were mappable to the dog genome (Fig. 4a). There was no systematic difference between both protocols (*p* = 0.5936). Furthermore, we investigated how many mapped reads were informative in respect to exonic regions of the genome. The fraction of reads mapping to exonic regions was between 14 and 17.07% (Fig. 4b). On average, 1.55% more reads using the new protocol were assigned to exonic regions (p = 0.0323). Finally, the gene body coverage was checked to determine biases at 3′- or 5′-end of expressed genes (FPKM > 10). The coverage profiles were very flat for all libraries, indicating even gene body coverage (Fig. 4c). Nevertheless we observed a small bias at the 3′-end in the libraries of the old protocol. In conclusion, RNA extracted using the new focused ultrasonication based method yields RNA that performs comparably to the old protease-based extraction techniques in NGS applications.

Concluding, the vastly increased yield combined by its compatibility with qPCR and NGS makes the new extraction method a preferential choice for extraction of RNA from limiting amounts of FFPE clinical specimen.

## Discussion

FFPE tissue archives constitute a vast treasury of valuable samples for biomedical research. Thus far however, the extraction of RNA from FFPE tissues has proved challenging due to chemical crosslinking of RNA with proteins as well as RNA fragmentation, both of which heavily impact on RNA quantity and quality for downstream analysis. When, additionally to these difficulties, sample size become very small, e.g. due to the need to use LCM to isolate specific subpopulations of cells, it has been very difficult to recover enough RNA that is amenable for proper analysis using RT-qPCR or NGS.

Encountering this problem, we found that commonly used RNA isolation protocols were mostly inefficient due to insufficient initial steps of tissue disruption using proteinase K, which most of the time left behind still macroscopically visible pieces of tissue. To overcome this bottleneck, we successfully adapted an isolation protocol for paraffinised FFPE samples that uses focused ultrasonication for efficient tissue disruption to our deparaffinised, stained and laser-capture-microdissected samples.

Using this approach, we could show that total RNA yields could be increased 8–12-fold compared to the commonly used proteinase K-based tissue disruption techniques (Fig. 3). More importantly, RNA extracted with this new approach proved to be qualitatively at least equal if not superior to RNA extracted using the old approach, as RT-qPCR Ct values were on average 2.3-fold lower using the new method (Fig. 3b). This was surprising, as the RIN values did not differ significantly between the old and the new extraction method (Fig. 3c). As the most important determinants for RNA integrity in FFPE samples seem to be the fixation and storage steps, and can not be greatly influenced by the method of extraction [2], we hypothesize that the reason for this better performance is possibly lower amounts of protein-RNA crosslinks present when using the new method. This could be caused by better sample accessibility for proteinase K, which would explain a better ‘usability’ of RNA for cDNA generation. Finally, we demonstrate that the RNA extracted using the new method performs well in NGS and is thus amenable for analysis using this technique (Fig. 4).

## Conclusions

Using this optimised RNA extraction protocol, analysis of limiting samples derived from LCM extracted deparaffinised and stained FFPE tissue samples becomes technically feasible. We thus envisage that the application of this protocol can have a tremendous positive impact on the feasibility of RNA expression studies on archival patient-derived samples. The possibility to study gene expression signatures in specific sections of archival FFPE tissue, which often entail large amounts of highly relevant clinical follow-up data, unlocks a new dimension of hitherto difficult-to-analyse samples which now become amenable for investigation. Importantly, this approach is perfectly translatable to human tissue specimen and different pathologies, and we hope to enable a variety of studies to come to a successful completion using our RNA extraction and NGS-protocols.

## Accession numbers

The sequence data of this study have deposited in the European Nucleotide Archive with the primary Accession Code PRJEB20761.

## Additional file

**Additional file 1: Table S1.** Total extracted RNA amounts and RIN values for all samples. Table listing the total extracted RNA amounts and RIN values for all samples included in the study. N.d. = not detectable.

## Abbreviations

FFPE: formalin-fixed paraffin embedded
LCM: Laser-capture microdissection
RT-qPCR: reverse-transcription quantitative PCR
CAS: cancer-associated stroma
NGS: next-generation RNA sequencing
DEPC: diethyl pyrocarbonate
PEN: polyethylene naphtalate
