# Supplementary material for: An optimised protocol for isolation of RNA from small sections of laser-capture microdissected FFPE tissue amenable for next-generation sequencing
Source: BMC Mol Biol. 2017 Aug 23;18:22. doi: 10.1186/s12867-017-0099-7 (PMC5569520; doi:10.1186/s12867-017-0099-7)
Supplement: Supplementary file 1 — Additional file 1: Table S1. Total extracted RNA amounts and RIN values for all samples. Table listing the total extracted RNA amounts and RIN values for all samples included in the study. N.d. = not detectable. [file 12867_2017_99_MOESM1_ESM.docx]

**Table S1:** Total extracted RNA amounts and RIN values for all samples

| Sample# | Old Normal | | Old CAS | | New Normal | | New CAS | |
| --- | --- | --- | --- | --- | --- | --- | --- | --- |
|  | RNA(ng) | RIN | RNA(ng) | RIN | RNA(ng) | RIN | RNA(ng) | RIN |
| 1 | 3.3 | 1.5 | 3.1 | 1.9 | 79.8 | 2.7 | 194.0 | 2.4 |
| 2 | 4.5 | 1.4 | 8.2 | 1.9 | 143.6 | 2.4 | 136.0 | 2.5 |
| 3 | 34.0 | 3.9 | 31.0 | 2.1 | 9.7 | 1.9 | 70.2 | 1.6 |
| 4 | 13.4 | 2.1 | 9.8 | 2.1 | 11.8 | 1.6 | 36.0 | 1.9 |
| 5 | 7.8 | 2.8 | 6.0 | 3.2 | 36.3 | 1.4 | 14.6 | 1.4 |
| 6 | 6.7 | 2.1 | 7.3 | 1.3 | 13.3 | 2.2 | 50.4 | 2.3 |
| 7 | 2.9 | n.d. | 5.2 | n.d. | 44.4 | 2.4 | 86.1 | 2.8 |
| 8 | 5.0 | n.d. | 4.4 | 2.5 | 39.9 | 2.8 | 30.0 | 2.6 |
| 9 | 6.5 | 2.5 | 17.3 | 2.3 | 35.1 | 2.7 | 103.2 | 2.0 |
| 10 | 5.7 | 2.4 | 8.9 | 2.2 | 46.2 | 2.9 | 58.2 | 2.9 |
| 11 | 18.2 | 2.3 | 1.7 | n.d. | 26.9 | 2.9 | 55.2 | 2.9 |
| 12 | 18.2 | 2.1 | 9.1 | 2.3 | 23.5 | 1.7 | 43.9 | 2.3 |
| 13 | 6.1 | n.d. | 12.4 | 2.2 | 17.0 | 1.8 | 44.5 | 1.6 |

Table listing the total extracted RNA amounts and RIN values for all samples included in the study. N.d. = not detectable.
